# Modulation of Endocannabinoid System Components in Depression: Pre-Clinical and Clinical Evidence

**DOI:** 10.3390/ijms23105526

**Published:** 2022-05-15

**Authors:** Uri Bright, Irit Akirav

**Affiliations:** 1 Department of Psychology, School of Psychological Sciences, University of Haifa, Haifa 3498838, Israel; uribr@hotmail.co.il; 2 The Integrated Brain and Behavior Research Center (IBBRC), University of Haifa, Haifa 3498838, Israel

**Keywords:** cannabis, cannabinoids, endocannabinoid system, depression, rodent models

## Abstract

Depression is characterized by continuous low mood and loss of interest or pleasure in enjoyable activities. First-line medications for mood disorders mostly target the monoaminergic system; however, many patients do not find relief with these medications, and those who do suffer from negative side effects and a discouragingly low rate of remission. Studies suggest that the endocannabinoid system (ECS) may be involved in the etiology of depression and that targeting the ECS has the potential to alleviate depression. ECS components (such as receptors, endocannabinoid ligands, and degrading enzymes) are key neuromodulators in motivation and cognition as well as in the regulation of stress and emotions. Studies in depressed patients and in animal models for depression have reported deficits in ECS components, which is motivating researchers to identify potential diagnostic and therapeutic biomarkers within the ECS. By understanding the effects of cannabinoids on ECS components in depression, we enhance our understanding of which brain targets they hit, what biological processes they alter, and eventually how to use this information to design better therapeutic options. In this article, we discuss the literature on the effects of cannabinoids on ECS components of specific depression-like behaviors and phenotypes in rodents and then describe the findings in depressed patients. A better understanding of the effects of cannabinoids on ECS components in depression may direct future research efforts to enhance diagnosis and treatment.

## 1. Introduction

Depression is one of the world’s most common psychiatric disorders, with a prevalence rate of 3.8%, according to the World Health Organization [1]. The lifetime prevalence of depression is as high as 20%, with a female-to-male ratio of about 5:2 [2]. Major depressive disorder (MDD) has been one of the leading causes of years lived with disability during the last three decades and it is also a major contributor to suicide deaths [1,3]. 

The Diagnostic and Statistical Manual of Mental Disorders (DSM-5), the guide used by health care professionals, states that the common feature of depressive disorders is the presence of sad, empty or irritable mood, accompanied by somatic and cognitive changes that significantly affect the individual’s capacity to function [4]. 

Depression is a complex phenomenon with many subtypes and many likely etiologies. There are multiple treatments with varying success rates, but the efficacy of currently used drugs is limited, particularly for preventing relapse and recurrence [5]. Selective serotonin reuptake inhibitors (SSRIs) and cognitive-behavioral therapy (CBT) are the two first-line treatments for depression [6]. SSRIs are among the most commonly prescribed drugs worldwide and are better tolerated than their predecessors, the tricyclic antidepressant family (TCAs); however, they have adverse side effects and when discontinued by the patient might cause withdrawal and rebound phenomena [7,8].

Many patients do not respond to SSRIs, or show intolerance to the drugs’ undesired effects [9]. About 60% of MDD patients continue to report residual impairments even after treatment with SSRIs [10], and around 33% of MDD patients develop resistance to antidepressant drugs [11]. Moreover, about 38% of patients suffer from at least one side effect, the most common of which include impaired sexual functioning, sleeping problems, and weight gain [12]. These negative side effects are common across antidepressant drug classes, and can withhold initiation of drug treatment and contribute to discontinuation of treatment [13,14]. Therefore, extensive efforts have been made to develop new approaches that treat depression with reduced side effects.

This partial success in treating depression is associated with our insufficient understanding of the underlying mechanisms of the disorder. In this sense, there is evidence to suggest that the endocannabinoid system (ECS) is impaired in MDD, providing a unique opportunity to identify potential diagnostic and therapeutic biomarkers. The ECS is a widespread neuromodulatory system involving a combination of endocannabinoids, enzymes, and cannabinoid receptors that help regulate numerous functions, including emotions and cognition.

A growing body of evidence suggests that the etiology of MDD may involve the ECS [15]. Specifically, it has been proposed that ECS deficits might have a depressive and anxiogenic effect on behavior, while elevation of ECS signaling can have antidepressant and anxiolytic properties [16,17]. Hence, some cannabis sativa plant compounds, which target the ECS, have been attracting great interest for their potential therapeutic use [18]. Recent measurements of public opinion suggest that people believe cannabis provides relief from depression and do not perceive it as harmful [19]. Due to the increase in the number of people that self-medicate with cannabis to relieve depressive symptoms, it is essential to determine whether cannabis is effective for managing depression.

Longitudinal studies have reported mixed evidence regarding the association between cannabis use and depression [20,21]. Some suggest that cannabis use may increase the risk for developing depression [22,23], while others found that cannabis users and nonusers were equally prone to develop depression [24,25]. Another longitudinal study suggests that MDD is associated with future initiation of cannabis use, hence suggesting self-medication [15].

Overall, it seems that depressed individuals may start using cannabis or increase the frequency of cannabis use as a way to “self-medicate” and relieve their symptoms; on the other hand, cannabis use may increase the risk for depression in heavy users who initiated their consumption in early adolescence [26,27]. Cannabis users who initiated early use and frequently used cannabis during adolescence might be at risk to develop cannabis use disorder (CUD) [28]. The estimated chances of becoming addicted to cannabis after lifetime exposure are 8.9% [29].

Cannabinoids are molecules that act on cannabinoid receptors type 1 and 2 (CB1r and CB2r) and can be divided into three broad categories: endogenous cannabinoids, synthetic cannabinoids, and plant-derived cannabinoids. The main endogenous cannabinoids are the signaling lipids N-arachidonoylethanolamine (anandamide, AEA) and 2-arachidonoylglycerol (2-AG). Synthetic cannabinoids are produced by academic laboratories or the pharmaceutical industry for research (e.g., HU-210, WIN 55,212-2, CP 47,497) or produced as popular recreational drugs [30,31]. They have a pharmacological effect by binding to CB1r and/or CB2r, with CB1 agonists responsible for the recreational effects of the synthetic cannabinoids; their effects are considered to be intense and faster than those observed with cannabis smoking, explained partly by the full agonist activity and high affinity for cannabinoid receptors [30]. The cannabis plant contains over 500 constituents, with the main compounds including delta-9-tetrahydrocannabinol (THC) and cannabidiol (CBD) [32]. THC is the main psychoactive compound in cannabis, which produces the “high” sensation and could lead to adverse consequences. CBD that is derived directly from the hemp plant exhibits no effects indicative of any abuse or dependence potential in humans [33]. The cannabis plant also contains chemicals such as alkaloids, terpenes, flavonoids, phenolic acids, etc., that may elicit physiological responses in humans, some of them anxiolytic [34].

The effects of the cannabis plant have been studied in both humans and rodents in order to elucidate the involvement of the ECS in the etiology and treatment of psychiatric disorders (for recent reviews: [35,36,37]). Furthermore, positive effects have been reported when using whole plant extracts, where the whole spectrum of cannabinoids and other bioactive and non-active compounds is present; this is called the “entourage effect” [38].

Human studies for treating different psychiatric disorders have mostly focused on the cannabis sativa plant and its main compounds THC, CBD or a combination of them [17]. In studies using rodent models, researchers are attempting to isolate new cannabinoid agonists to examine their potential therapeutic effects [39,40,41,42].

When considering the efficacy of cannabis and cannabinoids for depression (or any other neuropsychiatric condition), it should be taken into consideration that cannabis has multiple components. There is a high diversity across types and strains of herbal cannabis, and pharmacological differences across cannabinoids, but only a few studies in humans that have compared these differences [43]. Hence, assessing the relative effectiveness of different cannabis strains and different cannabinoids for diverse outcomes requires further research.

In this article, we will provide an overview of the neuromodulatory effects of cannabinoid compounds on different components of the ECS (such as receptors and ligands). While studies on the effects of the whole plant on depression are important, examining the differential effects of cannabinoids on ECS components may improve our diagnosis and enhance our treatment options. To that end, we review the current knowledge about the role played by the various components of the ECS in the etiology and treatment of depression in animal models and in humans.

In the following sections, we briefly describe the ECS and then review the effects of cannabinoids on ECS components in rodent models of specific depression-like behaviors and endophenotypes. We will review the literature about the effects of agonists and antagonists of cannabinoid receptors and then discuss the findings regarding CBD. Next, we describe the findings in human subjects, specifically, in subjects with a primary diagnosis that is not depression and in self-medicating subjects. Then we will review studies assessing alterations in ECS components in depressed patients (in endogenous ligands and cannabinoid receptors) and genetic variants of the ECS that are associated with depression.

## 2. Cannabis and the Endocannabinoid System (ECS)

Cannabis is the most commonly used addictive substance following tobacco and alcohol, and the number of cannabis users continues to increase [44,45]. Each strain of the cannabis plant consists of roughly 120 phytocannabinoids, the most studied of which are CBD and THC [46]. Much of the interaction of these phytocannabinoids with the mammalian nervous system is through the ECS. The main receptors of the ECS are CB1r and CB2r, cannabinoid receptors belonging to the category of G-protein-coupled receptors. CB1r is found primarily in the brain and CB2r is expressed mainly in peripheral organs, especially cells associated with the immune system [47], though it is also present in the brain [48]. Other non-CB1r/non-CB2r targets of cannabinoids include transient receptor potential vanilloid 1 (TRPV1), G-protein-coupled receptor 55 (GPR55), and peroxisome proliferator-activated receptors (PPARs). As mentioned above, the main endogenous ligands of CB1r and CB2r are AEA and 2-AG. AEA is a high-affinity, partial agonist of CB1r that is almost inactive at CB2r; 2-AG is a full agonist of both CB1r and CB2r, with moderate-to-low affinity; AEA and 2-AG also interact with TRPV1 and GPR55 [49,50,51].

AEA and 2-AG are produced at postsynaptic neurons and are lipophilic molecules that are synthesized “on demand” from membrane phospholipids. They are released immediately and without being stored in vesicles. The enzymes responsible for degrading AEA and 2-AG are, respectively, the fatty acid amide hydrolase (FAAH) and the monoacylglycerol lipase (MAGL). AEA is hydrolyzed in postsynaptic neurons by FAAH, thus terminating the AEA action at the time of its synthesis, whereas 2-AG is hydrolyzed in presynaptic neurons by MAGL, following CB1r activation.

CBD acts on several targets, including the serotonin 1A receptor (5-HT1A), GPR55 and TRPV1 [52,53], as well as CB1r and CB2r, with low affinity [54]. It is a GPR55-antagonist [53] and acts as a negative allosteric modulator of CB1r, modifying the power and efficiency with which endogenous cannabinoids activate the receptor [55], and as an inverse agonist at very high concentrations [56]. CBD also inhibits the metabolization of FAAH, which increases AEA tone; it has been suggested that this is the mechanism by which CBD activates CB1r [57]. THC is an agonist of both CB1r and CB2r, with lower affinity than several other synthetic cannabinoids such as WIN55,212-2, CP55940, and the endocannabinoid 2-AG, but with similar affinity as AEA [58]. The characterization of the mode of action of THC underlies a wide spectrum of pharmacological effects, which encompass euphoria, calmness, appetite stimulation, sensory alterations, and analgesia [59].

## 3. Studies of Depression in Rodent Models

Rodent models for depression do not represent the human condition in its entirety [60]; rather, they represent specific features of depression. In this way, they achieve a better understanding of one critical biological function of the disease to help translate it to the human condition [61,62]. They also provide a crucial approach to examine neural circuitry and molecular and cellular pathways in a controlled environment.

Widely used models for depression are chronic mild stress (CMS) or chronic unpredictable mild stress (CUMS), chronic social defeat stress, learned helplessness, and early life stress (ELS); all cause significant changes in behavior, brain functioning and physiology. CMS/CUMS comprises a series of trials, such as day and night reversal, tail clipping, and water or food deprivation, for a period of 3 weeks or more. The model uses repeated stressors to avoid the stress adaptation that may occur following a single repeated stimulation. The chronic social defeat stress comprises of repeatedly exposing naïve male mice to aggressor mice. In learned helplessness, animals manifest a low intention to escape in an environment of uncontrollable and unpredictable injury stimulation. In ELS, adverse events in early life substantially affect the development of psychiatric illnesses in late life, such as depression [63,64].

The behavioral outcome measured in rodents is usually despair-like behavior and anhedonia. Anhedonia, a loss of interest in things that were once pleasurable, is a common symptom of depression as well as other mental health disorders. These tests are also used to measure the antidepressant potential of new compounds. In the forced swim test (FST) and the tail suspension test (TST), a rodent is exposed to a stressful and inescapable situation (swimming in the FST and suspension by its tail in the TST), and the duration of its immobility is measured. The FST is based on the assumption that when placing an animal in a container filled with water, it will first make efforts to escape but eventually will exhibit immobility that may be considered to reflect a measure of behavioral despair. These tests have good predictive validity and are able to identify drugs that may be effective in depressed patients [65]. Another frequently used measure is the saccharine/sucrose preference test: the consumption of sweetened water or choosing between sweetened water and plain water in order to measure sensitivity to reward. Decreased consumption of palatable solutions or decreased preference are considered to reflect the condition of anhedonia [66].

Most preclinical research on depression has been performed on male rodents [67]. This is despite the fact that, in humans, depression is more prevalent in women than men [68]. Furthermore, men and women in most cases differ at baseline and in their responses to stress and drugs [60], which emphasizes the need to study both sexes.

## 4. The Effects of Cannabinoids on ECS Components in Rodents

### 4.1. CB1r

CB1r in the central nervous system is distributed densely in limbic regions associated with stress and cognition, including the nucleus accumbens (NAc), hippocampus, amygdala, and paraventricular nucleus (PVN) of the hypothalamus. CB1r is abundant in the prefrontal cortex (PFC), as well as in areas involved in pain transmission and modulation; in motor regions such as the basal ganglia and cerebellum; and in glial cells and the periphery [69,70]. In this section, we review the effects of cannabinoids on CB1r in rodent models for depression.

#### 4.1.1. Pre-Clinical Studies of CB1r Knockout and Antagonism

A number of studies have indicated a major role for CB1r in the etiology of depression, and it is estimated that its intact function is essential for a healthy mood [71]. Several studies have shown that CB1-knockout or knockdown-mice are prone to depressive-like behavior [72,73]. For example, CB1-knockout mice that were exposed to CUMS exhibited an augmented susceptibility to develop an anhedonic state, suggesting increased depressive-like behavior [73]. In a more recent study, exposure to the chronic social defeat model selectively potentiated excitatory transmission in cholecystokinin glutamatergic neurons in the basolateral amygdala (BLA) and D2 medium spiny neurons in the NAc via reduction of presynaptic CB1r [74]. Importantly, knockdown of CB1r in this circuit increased stress susceptibility, and the CB1r agonist administered to the NAc had antidepressant-like effects. This suggests that downregulating CB1r in this circuit is essential for stress-induced depression [74].

Chronic CB1r-antagonists can also result in a depressed mood. For example, 21-day intraperitoneal (i.p.) treatment with the CB1r-antagonist rimonabant (10 mg/kg) increased immobility time in the FST (i.e., elevated levels of despair-like behavior) and decreased sucrose preference (i.e., anhedonia) [75]. A summary of the effects of CB1 antagonists on depression-like behavior in rodents is presented in Table 1.

In addition, acute or chronic AM251 administration (0.3, 1 mg/kg, i.p.) to rodents exposed to stress-induced depression can inhibit the antidepressant-like effects induced by other substances and methods, such as AEA [83], repetitive transcranial magnetic stimulation [84], the synthetic non-selective cannabinoid receptor agonist WIN55,212-2 [85], the MAGL inhibitor JZL184 [86], CBD [87], the FAAH inhibitor URB597 [86] and the AEA reuptake inhibitor AM404 [88]. Acute administration of rimonabant (3 mg/kg, i.p.) prevented a decrease in immobility time in the FST induced by URB597, AM404 and CP55,940 [89]. URB597 inhibits AEA degradation and enhances AEA availability in the synapses, and thus functions as an indirect agonist of CB1r. AM404 is another enhancer of AEA, as it acts as an AEA reuptake inhibitor. CP55,940 is a potent and non-selective synthetic cannabinoid agonist. A summary of the effects of CB1 antagonists co-administered with cannabinoid agonists on depression-like behavior in rodents is presented in Table 2.

Intracerebral injections of AM251 showed similar effects; AM251 (0.8 μg) microinjection to the NAc inhibited antidepressant-like effects induced by the antidepressant phenylglycine derivative (RS)-2-chloro-5-hydroxyphenylglycine in mice that underwent the chronic social defeat stress [90]; intracerebroventricular (i.c.v.) injection of AM251 (1 μg) prevented anti-depressant effects induced by URB597 [76]. AM251 (0.28 ng) microinjection to the PFC augmented depressive-like behaviors induced by CUMS [77] and prevented the therapeutic-like effect of URB597, which decreased immobility time in the FST [91]. AM251 (0.01 ng) to the CA1 region of the hippocampus induced despair-like behavior in the FST [78,79].

Even though the majority of research shows that CB1r-antagonists enhance depressive-like behaviors, several studies have found the opposite; two-time oral administration of rimonabant (3 and 10 mg/kg) reduced immobility time in the FST in naive mice [82], suggesting an antidepressant effect; chronic oral administration of rimonabant (10 mg/kg) for 5 weeks reduced immobility time in CMS mice [82]; acute AM251 (0.3, 0.5, 1 and 10 mg/kg) reduced immobility time in the FST in mice [80] and 0.3, 0.5 and 1 mg/kg decreased immobility in the TST [80]. AM251 (0.25 mg/kg, i.p.) also augmented the antidepressant effects of tianeptine (a tricyclic antidepressant) and agomelatine (an atypical antidepressant) in mice [92]. In addition, intra-BLA microinjection of AM251 (0.01μg/ 0.5μL) reduced immobility time in the FST in rats [81].

It is interesting that rimonabant had opposite effects when administered orally, compared to i.p. and microinjections [75,82]. This suggests that different mechanisms may mediate its effect when ingested and not injected systemically. As for AM251, acute i.p. administration decreased depression-like behavior [82]. However, when administered following AEA treatment, acute AM251 blocked the antidepressant effects induced by AEA [83]; similarly, when chronically co-administered with other treatments (e.g., CB1r agonists), it blocked their therapeutic-like effects on behavior [79,80,84,85,86]. This emphasizes the complex mechanisms underlying the effects of CB1r-antagonists on depression. This complexity is further stressed by the dose-dependent, biphasic effects of CB1 ligation found in multiple studies regarding different effects on the ECS [93,94,95,96].

Taken together, these results propose that CB1r deficiency represents a model for depressive-like disorders [97], but the diversity of these findings suggests that more study is needed to fully understand the role played by CB1r-antagonism in depression.

#### 4.1.2. Pre-Clinical Studies of CB1 Receptor Agonism

AEA generally has antidepressant properties. Multiple studies have shown that chronic i.p. injection of the FAAH-inhibitor URB597 (0.2, 0.3, 0.4, 5.8 mg/kg), which increases AEA levels, prevents depressive-like behaviors induced by different models and methods, such as CUMS [98], adolescent THC exposure [99], CMS [100] and chronic constriction injury (CCI) that induces neuropathic pain and depression-associated behavior [101]. We showed that 14-day administration of URB597 (0.4 mg/kg, i.p.), the MAGL inhibitor JZL184 (2 mg/kg; i.p) or the CB1/2 receptor agonist WIN55,212-2 (1.2 mg/kg, i.p.) during late adolescence decreased depressive-like behaviors induced by ELS in male and female rats [86,102,103]. However, when administered at mid-adolescence, the same dose of URB597 did not prevent the deleterious long-term effects of ELS exposure on depression-like behavior in males and females and induced long-term, depressive-like behavior by itself in non-stressed rats [103]. This suggests that URB597 may have deleterious or ameliorating effects on behavior, depending on the developmental time window of treatment (i.e., mid- or late adolescence) [103].

Both chronic and acute URB597 (0.3 mg/kg) and AM404 (5 mg/kg) prevented depressive-like behaviors in the FST induced by severe electric shock [40] and nicotine abstinence [88], respectively. The same effect was seen in naive rats, in which URB597 (0.1, 0.3, 1, 3.2 mg/kg, i.p.), AM404 (0.3, 1, 3 mg/kg, i.p.), CP55,940 (0.1 mg/kg, i.p.), and the CB1r-agonist oleamide (10, 20 mg/kg, i.p.) decreased FST immobility time, supporting the antidepressant-like effects of these compounds [89,104,105,106]. Oleamide, a fatty amide derived from oleic acid (5 mg/kg, i.p.), also augmented the antidepressant-like effects of the atypical antidepressant tianeptine in the FST [78]. The CB1r synthetic agonist arachidonyl-2′-chloroethylamide (ACEA; 10 mg/kg, i.p.) increased sucrose consumption in post-stroke depression rats, suggesting decreased anhedonia [107]; post-stroke depression is one of the most common psychological consequences of stroke.

I.c.v. injection of URB597 (5 and 10 ng) prevented depressive behaviors that were induced by methamphetamine in mice [76]. Despair-like behavior also was decreased in mice by i.c.v. administration of URB597 (0.05, 0.1, 1, 5, 10 μg), AM404 (0.1, 1, 5, 10 μg), and AEA (1, 5, 10, 20 μg) [108], and by microinjection of URB597 (0.01, 0.1, 1 nmol) to the ventromedial PFC [109]. Microinjection of URB597 (0.01 μg) to the PFC in rats reduced FST immobility time [91]. No effect on FST performance, however, was seen after microinjection of URB597 (0.5, 1 μg) to the dentate gyrus of the hippocampus. However, administration of the CB1/CB2-agonist HU-210 (1, 2.5 μg) to the same region decreased immobility time in the FST [110]. A summary of CB1-mediated effects of cannabinoids on depression-like behavior in rodents is presented in Table 3.

All things considered, the data propose that augmenting ECS signaling via CB1r may be a novel approach to decrease depression-like behavior and that the use of CB1r antagonists warrants caution. Specifically, FAAH inhibition, which enhances AEA-mediated CB1r signaling, has been suggested to generate a more specific and beneficial spectrum of biological effects than those caused by direct CB1r agonists [111,112].

### 4.2. CB2r

CB2r was discovered at the beginning of the 1990s, and at first, it was assumed to be present mainly in peripheral and immune tissues [113]. However, its presence has been observed in some subsets of neurons in the brain and thus this receptor likely participates in the modulation of neurotransmission [114]. CB2r is mainly studied in pain and inflammation, yet a growing number of studies provide evidence of a potential role of CB2r in the etiology of depression [115]; the main endogenous ligand for CB2r is 2-AG [116].

#### 4.2.1. Pre-Clinical Studies of CB2 Knockout and Antagonism

The outcomes of CB2-antagonists administration seem to be dose-dependent. On the one hand, it may enhance depressive-like behaviors, or invert the antidepressant effects of CB2r-dependent treatments. On the other hand, it may facilitate antidepressant effects induced by other treatments. For example, the CB2-inverse agonist AM630 (1 mg/kg i.p.) blocks the antidepressant effects in the FST induced by CBD in diabetic rats [87], but works as an antidepressant when administered at a lower dose (0.5 mg/kg, i.p.) [104]; diabetic patients are two to three times more likely to develop depression and diabetic rats demonstrate depression-like behaviors [117]. When administered acutely at a low dose (0.25 mg/kg, i.p.) in mice, AM630 augments the antidepressant effects of the tricyclic antidepressant imipramine, the SSRI escitalopram, the norepinephrine reuptake inhibitor reboxetine, and the atypical antidepressants agomelatine and tianeptine [92,118].

#### 4.2.2. Pre-Clinical Studies of CB2 Agonism

Several studies report that CB2r agonists have antidepressant properties. For example, the CB2-full agonist β-Caryophyllene (BCP) ameliorated depressive-like behaviors (i.e., reduced immobility time in the TST and FST) when acutely administered i.p. (50 mg/kg) in mice [119] and chronically administered (25, 50, 100 mg/kg) in rats that were subjected to daily restraint stress [120]. Chronic oral administration of BCP (10 mg/kg) was effective in reducing immobility in the TST in diabetic mice [121].

An acute low dose of the CB2 agonist JWH 133 (0.25 mg/kg, i.p.), increased the antidepressant effects of the tricyclic antidepressant imipramine, the SSRI escitalopram and the norepinephrine reuptake inhibitor reboxetine in mice [122], while higher doses (0.5, 1 mg/kg, i.p.) had similar effects on their own [104]. JWH133 significantly decreased anhedonia (i.e., increased sucrose consumption) when injected i.p. (5 mg/kg) for 7 days or when microinjected (3 μg) acutely into the ventromedial hypothalamus of post-stroke depression rats [107].

The CB2-agonist GW 405833 has been mainly studied as a treatment for pain, and was found to reverse depressive-like behaviors induced by chronic constriction injury (CCI) in rats (30 mg/kg, i.p.) [122]. Another study found that CB2 agonists and overexpression of CB2 were correlated with decreased depressive-like behavior in transgenic mice, as evidenced by the FST and the novelty-suppressed feeding test [123]; the novelty-suppressed feeding test is sensitive to chronic, but not acute, antidepressant treatment, and is assumed to mirror the effects of antidepressant treatment in human patients.

Overall, compounds used to activate CB2r seem to intensify the antidepressant-like effects induced by other drugs. This suggests that CB2r is involved in depression-related behaviors through interactions with other systems that modulate these responses (e.g., serotonergic).

### 4.3. GPR55

The GPR55 receptor was cloned in 1999 [124] and was later characterized as part of the ECS, as it binds AEA and 2-AG as well as THC and CBD [50,51]. There is evidence that GPR55 plays an important role in depression; a 7-day intravenous (i.v.) treatment with the GPR55-agonist O-1602 decreased despair-like behavior in female rats subjected to a 14-day corticosterone treatment [125]. A 10-day chronic social defeat stress lowered hippocampal GPR55 levels in mice that were susceptible to the model (i.e., showed elevated levels of depression and anxiety), but not in resilient mice. Interestingly, O-1602 treatment (10 mg/kg, i.p.) during chronic social defeat stress decreased these behaviors [126]. Compared to a control group, the learned helplessness model decreased GPR55 mRNA levels in the lateral habenula and the amygdala, with no effects in the hippocampus and medial PFC [127]. To summarize, these studies suggest that exposure to stress-induced depression results in decreased levels of GPR55 in a region-dependent manner and that the GPR55 agonist has antidepressant effects on behavior.

### 4.4. TRPV1

TRPV1 is a nociceptive receptor that has been thoroughly studied in the context of pain [128]. Considering the large comorbidity of pain and depression [129], it is not surprising that there are interesting findings regarding the role of TRPV1 in depression. It is generally assumed that TRPV1-agonists induce depressive behavior, and that TRPV1-antagonists may provoke the opposite effect [130].

Three injections (2.5, 5 mg/kg, i.p.) of AA-5-HT (a dual blocker of FAAH and TRPV1) reduced immobility time in stressed rats [131]. AA-5-HT attenuated despair-like behavior in rats, also when microinjected (0.25, 0.5 nmol) into the PFC [109,132]. TRPV1 mRNA levels in the medial PFC of mice that underwent the learned helplessness model of depression were significantly lower than in control mice [127].

### 4.5. CBD

CBD has multiple key targets, including cannabinoid receptors, 5-HT1A receptors, and neurogenesis factors, and hence is addressed separately in this section. Studies have shown its potential to treat depressive-like behaviors; for example, acute treatment with CBD (200 mg/kg, i.p.) reduced immobility time in the FST in mice [133]; a 7-day treatment in adolescent rats, adult rats and mice (10, 30, 100 mg/kg, i.p., respectively) reduced FST immobility time [134,135]. Moreover, a sub-chronic administration of CBD (30 mg/kg, i.p.) reduced despair-like behavior in diabetic mice [136]; a chronic, 28-day administration of CBD (10 mg/kg, i.p.) elevated sucrose intake in CUMS rats [137]. CBD was effective in lowering depression behaviors in two rat strains genetically modified for depression research; acute oral CBD (30 mg/kg) reduced immobility time in the FST and elevated saccharine consumption in male and female Wistar Kyoto rats and reduced immobility in males of the Flinders Sensitive Line [39,138].

Acute CBD (10 mg/kg, i.p.) lowered FST immobility time in mice, both 30 min and 7 days after administration [139,140,141]. A lower dose of CBD (7 mg/kg, i.p.) was as effective in lowering FST immobility time when co-administered with ineffective doses of the TCA desipramine, the SSRI fluoxetine, and the DNA-methylation inhibitors AzaD and RG108 [139,141]. The short-term antidepressant effects of CBD were associated with increased medial PFC expression of synaptophysin, PSD95, and brain-derived neurotrophic factor (BDNF), as well as elevated hippocampal BDNF [142]. Chronic CBD (15 mg/kg, i.p.) treatment also increased BDNF levels in the amygdala; a higher dose of CBD (30 mg/kg, i.p.) produced antidepressant-like effects in the FST when administered acutely and chronically [142]. Both lower (10 mg/kg, i.v.) and higher doses (100 mg/kg, oral) had antidepressant properties in CMS mice, in association with increased BDNF and synaptophysin mRNA in the medial PFC and hippocampus [143]. In addition, microinjection of CBD (15, 30, 60 nmol) to the pre-limbic division of the medial PFC of neuropathic pain-mice resulted in lower despair-like behavior in the FST [144].

Although CBD interacts with many ECS receptors (including those for CB1, CB2, TRPV1, and GPR55), studies of its antidepressant properties have focused mainly on the serotonergic 5-HT1A receptor as the main receptor that mediates these effects: Acute CBD (30 mg/kg, i.p.) reduced immobility in the FST in naive mice, an effect that was blocked by the 5HT1A-antagonist WAY100635 [145]; 7-day administration of CBD (50 mg/kg, i.p.) improved sucrose intake in mice that underwent the olfactory bulbectomy model of depression; both antidepressant-like effects and enhanced cortical 5-HT/glutamate neurotransmission induced by CBD were prevented by 5-HT1A receptor blockade [146]; similarly, microinjection of CBD to the ventromedial PFC (10, 30, 60 nmol to the prelimbic subregion; 45, 60 nmol to the infralimbic subregion) reduced FST immobility time, an effect that was blocked by pretreatment with WAY100635; the CB1-antagonist AM251 also blocked the effects of CBD [147]. Interestingly, a study of the anxiolytic effects of chronic CBD (30 mg/kg, i.p.) identified a mediating role for CB1r and CB2r but not for 5HT1A receptor. Taken together, these studies suggest that the effects of CBD on anxiety and depression may be mediated by different mechanisms [148]. A summary of the effects of CBD on depression-like behavior in rodents is presented in Table 4.

## 5. The ECS in Human Studies of Depression

There is accumulating preclinical evidence that targeting the ECS could potentially benefit patients suffering from depression [149]. However, epidemiological and clinical studies do not provide strong evidence to support that cannabis can be used as an antidepressant [150].

In reviewing the literature, we found very few studies where cannabinoids were used to treat depression with well-designed, randomized control trials (RCTs). Yet there are other strong indications from human studies that encourage further research. The potential role for the ECS in depression comes from a series of studies indicating that the CB1r antagonist rimonabant is associated with the development of severe adverse effects, including depression and suicide [151]. Clinical observations showed that cannabis stimulates appetite (the “munchies”) [152]. Rimonabant was developed as an anti-obesity treatment. A meta-analysis conducted in 2007 concluded that 20 mg/day of rimonabant increases the risk of depressive symptoms [153]. A later study, however, found that rimonabant in the same dosage had no effect on mood [154]. These findings are in line with an FDA report about the safety of rimonabant, which stated that 26% of the subjects given 20 mg rimonabant daily later developed psychiatric symptoms, compared to 14% of those given placebo [154].

### 5.1. Subjects with a Primary Diagnosis That Is Not Depression

There are no published RCTs that examined the direct effect of THC or CBD on depressive symptoms [155]. However, many published RCTs have examined the effects of THC or THC:CBD on other conditions, such as pain and multiple sclerosis (MS) [156]. These RCTs did not find improvement in depression symptoms in these patients compared to placebo. For example, Nabiximols (Sativex; an equal mix of THC and CBD) produced no effect on symptoms of depression in people with MS or with chronic pain due to cancer [157,158]. A small RCT that examined the effects of CBD alone in chronic pain, also found no change in depressive symptoms [159].

It is important to note that these RCTs did not include subjects with a primary diagnosis of depression (i.e., depression was assessed indirectly); that the patients’ self-reported depression scores were already low [160,161,162]; and that psychiatric diagnosis was listed as an exclusion criterion in some of the studies [157,158]. In total, this makes it difficult to extrapolate these outcomes to people with clinical depression.

However, there are studies that support an antidepressant effect of cannabinoids when depression was assessed directly, although not the primary diagnosis. In one study, a cross-sectional, longitudinal, and experimental design on individuals with social pain showed that marijuana use predicted lower levels of later depression among participants who were lonely, and that those individuals who used marijuana relatively frequently were less likely to have experienced a DSM-IV major depressive event during the previous 12 months [163].

A similar result was observed in a study that examined the effects of prolonged CBD administration on psychological symptoms and cognition to a community sample of regular cannabis users; oral CBD reduced depressive- and psychotic-like symptoms and improved attentional switching, verbal learning, and memory. Moreover, CBD was well tolerated with no reported side effects [164]. Similarly, in a randomized, double-blind, inpatient trial, nabiximols was used as an agonist therapy for reducing the severity and time course of cannabis withdrawal and for retaining participants in withdrawal treatment; nabiximols suppressed withdrawal-related irritability, cravings, and significantly reduced depression [165].

### 5.2. Self-Medication Studies

There is evidence that pharmacological interventions (specifically, SSRIs) may be effective in treating depression at the population level, but may not always be visible at the patient level [155]. The small effect size of SSRIs [17] together with their adverse side effects, means that some depressed individuals often seek alternative treatments. As a result, patients with depression are increasingly using medicinal cannabis products to relieve their symptoms [155].

Some reports of depressed patients self-medicating with cannabis demonstrate lower levels of depressive symptoms and improved sleep [166,167,168]. However, other reports show adverse effects, as depressed patients who self-medicate with cannabis demonstrate increased mental health problems and lowered improvement in depression symptoms and suicidal ideation [169].

In a longitudinal, cross-sectional study, medicinal cannabis users reported reduced depression and improved quality of life compared to a control group that was considering (but had not yet initiated) medicinal cannabis use [170]. In an observational study, medicinal cannabis use reduced depressive symptoms in clinically depressed populations [155]; specifically, medicinal cannabis use was associated with better sleep, quality of life, and less pain. Moreover, the group that initiated cannabis use during the follow-up period demonstrated fewer depressive symptoms compared to a control group that never initiated cannabis use [155].

To correctly interpret the therapeutic potential of cannabis, it is important to restrict and separate the cannabis effects reported under recreational consumption compared to clinical trials under medical supervision. Although many people report using cannabis to manage a large variety of medical conditions, including depression, the gathering of information regarding self-medication makes it hard to draw firm quantitative conclusions about the effectiveness of treatment.

### 5.3. ECS Components Altered in Depression

Compelling evidence for the involvement of the ECS in depression comes from studies assessing alterations in ECS components in depressed patients. Elucidating the effects of depression on different targets of the ECS is important because the ECS is involved in eliciting potent effects on neurotransmission, neuroendocrine, and inflammatory processes, which are known to be disturbed in depression.

#### 5.3.1. Endogenous Ligands

Accumulating data suggest that depression is strongly associated with deficient endocannabinoid signaling [171], and hence provide compelling evidence for the involvement of the ECS in the etiology of depression and a rationale for activating the ECS to relieve depression. For example, serum levels of AEA and 2-AG were found to be decreased in depressed patients [172]. Interestingly, the reduction in 2-AG serum levels was negatively correlated with the duration of depressive episodes [173]. Another study found indications of a deficit in peripheral endocannabinoid activity: basal serum concentrations of AEA and 2-AG were significantly decreased in women with MDD relative to matched controls [172]. Other studies have shown increased levels of endocannabinoids following treatment; plasma levels of oleoylethanolamide, AEA, and 2-AG were increased in patients with depression treated with SSRIs compared to a non-depressed control group [174]. Similarly, in men and women patients with MDD, physical exercise elevated plasma levels of AEA and 2-AG; the authors suggested that endocannabinoids may contribute to the antidepressant effects of exercise in MDD [175,176]. Antidepressant treatment by electroconvulsive therapy (ECT) elevated AEA and to some extent 2-AG levels in the cerebrospinal fluid [177]. These studies indicate that the ECS is modulated by effective antidepressant treatment.

#### 5.3.2. CB1r

Studies have found indications of increased CB1r availability in depression. Concentrations of CB1r and CB1rmediated stimulation of G proteins in the PFC were found to be increased in subjects with major depression who had died by suicides relative to controls [178,179]. Similarly, treatment with SSRIs decreased expression of CB1r in the anterior cingulate cortex of postmortem MDD patients [180]. However, another study did not find changes in CB1r protein expression in depressive subjects compared to controls [173]. Increased CB1r availability in depression may be a compensation response to low AEA levels, as suggested in post-traumatic stress disorder (PTSD) [112].

It should be noted that chronic direct activation of CB1r downregulated CB1r [181,182], which may in turn result in a depression-like phenotype in certain individuals [75,183].

Taken together, the data suggest that enhancing endocannabinoid signaling may serve as an antidepressant; CB1r blockade produces depression; and chronic direct activation of CB1r may produce region-dependent CB1r desensitization and down-regulation that is associated with depression [21].

### 5.4. Genetic Studies

Several studies have reported associations between genetic variants of the cannabinoid receptor type 1 and type 2 genes (CNRs; CNR1 and CNR2) and a susceptibility to develop depression. However, such studies reported conflicting findings [184]. Genetic variants of CNRs can affect gene transcription (and, thereby, protein expression and biologic function) of these cannabinoid receptors [185]. ECS-related polymorphic gene variant alterations have been reported, which may have both diagnostic and therapeutic implications.

#### 5.4.1. CNR1

The interaction between specific genetic variations in CNR1 and the vulnerability to depression has recently gained great interest. In a population of opiate-dependent outpatients remitted under stable methadone treatment, subjects with one single nucleotide polymorphism (SNP) of the CNR1 (named rs2023239) had a lower prevalence of lifetime MDD [186]. However, two other studies found no relation between CNR1 microsatellite polymorphisms and depressive disorders [187] or between CNR1rs1049353 and MDD [188,189]. Another piece of evidence against the relationship between depression and CNR1 is a recent meta-analysis, which assessed the relation between CNR1 and CNR2 polymorphisms and depressive disorder susceptibility. This meta-analysis did not find a significant association of the CNR1rs1049353 SNP with depressive disorders [184].

#### 5.4.2. CNR2

Some studies indicate that dysfunctional CB2r can contribute to greater sensitivity to childhood trauma, a risk factor for depression; specifically, the CNR2 R63Q polymorphism was associated with anxious and depressive phenotypes following childhood trauma [190]. In support, the expression of CNR2 Q63R was also found to be higher in Japanese depressed patients [191] and alcoholics [192] (alcoholism is in high comorbidity with MDD [193]). One study reported a higher incidence of the CB2 allele Leu133Ile for bipolar disorder patients [194]. A recent meta-analysis found a significant association of CNR2rs2501432 with depressive disorders [184]; also, in the dorsolateral PFC of suicide victims, CB2 gene expression was 33% lower, but their levels of CB2 protein were higher, when compared to a control group. This difference might stem from a compensatory mechanism that controls gene half-life and protein turnover [195].

To summarize, genetic variations in CNR2 are associated with the vulnerability to depression; relating this marker to depression-associated brain dysfunction may potentially improve the diagnosis and treatment for depression.

#### 5.4.3. FAAH

In the aforementioned study that showed an association between CNR2 R63Q polymorphism and depression, the researchers also found that dysfunctional FAAH could contribute to greater sensitivity to childhood trauma [190]; specifically, the FAAH rs324420 polymorphism (i.e., C385A) was associated with anxious and depressive phenotypes following childhood trauma [196]. The same polymorphism was higher in MDD patients and bipolar disorder patients [189]. In a study in cannabis users, greater past-year cannabis use and FAAH rs324420 genotype predicted poor sleep quality, which was mediated by depressive symptoms. Moreover, participants with higher cannabis use and depressive symptoms reported more impaired sleep [197].

#### 5.4.4. GPR55

In a study that evaluated alterations of GPR55 in suicide victims compared to corresponding controls, GPR55 gene expression was 41% lower in the dorsolateral PFC of suicide victims, and GPR55 protein in these subjects were the same as in the control group [195]. The link between suicide and mental disorders (in particular, depression and alcohol use disorders) is well established, and dysfunctions in the dorsolateral PFC of patients who attempted suicide are associated with impaired executive functions and increased impulsivity [198].

To summarize, evidence from longitudinal studies suggests that depression might increase cannabis use and perhaps vice versa. There is evidence of alterations to the genetic and ECS components in depression, suggesting that the ECS may be critically involved in the pathophysiology of depression.

#### 5.4.5. Caveats

There is a growing belief that cannabis and other cannabinoids are harmless drugs that can decrease anxiety and depression and induce relaxation. Accordingly, the use of medicinal cannabis and cannabinoids has recently been increasing. However, only a few registered drugs (usually containing CBD and THC) are of high quality [35]. In fact, in many of the above studies, the sources of cannabis are unknown or uncontrolled [199]. Moreover, several studies showed that the effects of cannabis on depression symptoms may be positive or negative, depending on the time course of administration; hence, although it was found that cannabis provided a brief relief, the long-term effects were worsening of symptoms [200]. This suggests that the short- and long-term effects of cannabis in depression should be taken under consideration. Additionally, we should bear in mind the potential risk for adverse events during cannabinoid usage. There are reports of increased risk of acute psychotic symptoms [201], and in young adults, chronic daily use of cannabis might generate cannabis dependence [202].

## 6. Conclusions

Rodent studies strongly suggest that activating the ECS produces antidepressant-like responses in a variety of behavioral tests. The effects are dependent on dosing, route of administration, and other factors, but the overall effect is that both direct and indirect activation of ECS components and CB1r in particular have an antidepressant potential, whereas deficits in ECS signaling may have depressive effects.

In humans, most studies addressed a different primary medical disorder (pain, MS) other than depression, with depression as a secondary condition. In these studies, cannabinoids had no effect on depression [17,150], but there is a lack of high-quality studies where depression is the primary target of cannabis treatment. Studies that examined patients with a primary diagnosis of depression included a small sample size and other methodological flaws [150]. In addition, most studies in human subjects did not compare the efficacy of cannabinoids with those of existing antidepressant agents. Therefore, high quality, large-scale RCTs in depressed patients are needed to assess the effectiveness and safety of cannabinoids and to compare it with placebo and standard treatments.

To conclude, the findings on the effectiveness of cannabis and cannabinoid compounds in depression reveal inconsistencies in the outcomes obtained in animal models compared to findings in depressed patients. By elucidating the effects of cannabinoids on ECS components, we can enhance our understanding of which targets the compounds hit, what processes they alter, and, eventually, which of these effects are needed for therapeutic efficacy.

Elucidating the role of the ECS in the etiology of depression and revealing the effects of different cannabinoids on the ECS in depression increase the probability of choosing cannabinoid compounds that will be effective treatments; this is imperative because understanding how different cannabinoid compounds work can help stratify clinical trials to focus them on those patients most likely to respond.

## Figures and Tables

**Table 1:** A summary of the findings regarding the effects of CB1 antagonists on depression-like behavior in rodents.

Drug	Administration	Animals	Stress	Model	Effect	Reference
AM251 (CB1 antagonist)	Acute, 1 μg, i.c.v.	Male NMRI mice	-	FST	Elevated immobility	[76]
	Acute, 0.28 ng, PFC microinjection	Male SD rats	CUS	FST	Elevated immobility	[77]
	Acute, 0.01 ng, HIPP microinjection	Male Wistar rats	Sleep deprivation	FST	Elevated immobility	[78]
	Acute, 0.3, 0.5 mg/kg, i.p.	Male NMRI mice	Foot shock	FST	Decreased immobility	[79]
	Acute, 0.3, 0.5 mg/kg, i.p.	Male NMRI mice	Foot shock	TST	Decreased immobility	[79]
	Acute, 0.3, 0.5, 1, 10 mg/kg, i.p.	C57BL/6 male mice	-	FST	Decreased immobility	[80]
	Acute, 0.3, 0.5, 1 mg/kg, i.p.	C57BL/6 male mice	-	TST	Decreased immobility	[80]
	Acute, 0.01 μg, BLA microinjection	Male SD rats	-	FST	Decreased immobility	[81]
Rimonabant (CB1 antagonist)	Chronic (21 days), 10 mg/kg, i.p.	Male SD rats	-	FST	Elevated immobility	[75]
			-	SPT	Decreased sucrose preference	[75]
	Acute (2 times), 3 mg/kg, 10 mg/kg, oral	Male SD and Wistar rats	-	FST	Decreased immobility	[82]
	Chronic (35 days), 10 mg/kg, oral	OF1 mice	CMS	FST	Decreased immobility	[82]

rTMS: repetitive transcranial magnetic stimulation; CUMS: chronic unpredictable mild stress; CMS: chronic mild stress; ELS: early life stress; CHPG: (RS)-2-chloro-5-hydroxyphenylglycine; TST: Tail suspension test; i.c.v.: intracerebroventricular; CMS: chronic mild stress; HIPP: hippocampus; NMRI: Naval Medical Research Institute; CCI: chronic constriction injury; NP: neuropathic pain; SD: Sprague–Dawley; SPT: sucrose preference test; SaPT: saccharine preference test.

**Table 2:** A summary of the effects of CB1 antagonists co-administered with cannabinoid agonists on depression-like behavior in rodents.

Drug	Administration	Animals	Stress	Treatment	Model	Effect	Reference
AM251 (CB1 antagonist)	Acute, 1 mg/kg, i.p.	Male Wistar rats	Streptozotocin (diabetic)	AEA	FST	Elevated immobility	[83]
	7 days, 1 mg/kg, i.p.	Male SD rat	CUMS	rTMS	FST	Elevated immobility	[84]
	3 days, 0.3 mg/kg, i.p.	Male SD rats	CMS	WIN55,212-2	FST	Elevated immobility	[85]
	14 days, 0.3 mg/kg, i.p. Acute, 1 mg/kg, i.p.	Male and female SD rats	ELS	JZL184 or URB597	FST	Elevated immobility	[86]
		Male Wistar rats	Streptozotocin (diabetic)	CBD	FST	Elevated immobility	[87]
	Acute, 5 mg/kg, i.p.	Male Long-Evans rats	-	AM404	FST	Elevated immobility	[88]
	Acute, 0.8 μg, NAc microinjection	Male C57BL/6J mice	Social defeat	CHPG	TST	Elevated immobility	[90]
	Acute, 0.28 ng, PFC microinjection	Male SD rats	-	URB597	FST	Elevated immobility	[91]
	Acute, 0.25 mg/kg, i.p	Male Albino Swiss mice	-	Tianeptine	FST	Decreasedimmobility	[92]
Rimonabant (CB1 antagonist)	Acute, 3 mg/kg, i.p.	Male Wistar rats	-	URB597, AM404, CP55,940	FST	Elevated immobility	[89]

rTMS: repetitive transcranial magnetic stimulation; CUMS: chronic unpredictable mild stress; CMS: chronic mild stress; ELS: early life stress; CHPG: (RS)-2-chloro-5-hydroxyphenylglycine; TST: Tail suspension test; i.c.v..: intracerebroventricular; CMS: chronic mild stress; HIPP: hippocampus; NMRI: Naval Medical Research Institute; CCI: chronic constriction injury; NP: neuropathic pain; SPT: sucrose preference test; SaPT: saccharine preference test; SD: Sprague–Dawley.

**Table 3:** A summary of CB1-mediated effects of cannabinoids on depression-like behavior in rodents.

Drug	Administration	Animals	Stress	Model	Effect	Reference
URB597 (FAAH Inhibitor	Chronic, 0.2 mg/kg, i.p.	C57BL/6J mice	CUS	FST	Decreased immobility	[98]
	Chronic, 0.3 mg/kg, i.p.	Female SD rats	Adolescent THC	FST	Decreased immobility	[99]
	Chronic, 0.3 mg/kg, i.p.	Female SD rats	Adolescent THC	SPT	Elevated sucrose preference	[99]
	Chronic, 0.3 mg/kg, i.p.	Male Wistar rats	CMS	SPT	Elevated sucrose preference	[100]
	Chronic, 5.8 mg/kg, i.p. 14 days (during mid-adolescence), 0.4 mg/kg, i.p.	Male Wistar rats	CCI injury (NP)	FST	Decreased immobility	[101]
		Male and female SD rats	ELS	FST	Decreased immobility	[86]
	14 days (during late-adolescence), 0.4 mg/kg, i.p.	Male and female SD rats	ELS	FST	Elevated immobility	[103]
	Acute, 0.3 mg/kg, i.p.	Male SD rats	Severe shock	FST	Decreased immobility	[40]
	Acute, 0.3 mg/kg, i.p.	Male SD rats	Severe shock	SaPT	Elevated saccharine preference	[40]
	Acute, 0.03, 0.1, 0.3 mg/kg, i.p.	Male Wistar rats	-	FST	Decreased immobility	[89]
	Acute, 0.1 mg/kg, i.p.	Male C57BL/6 mice	-	FST	Decreased immobility	[106]
	Acute, 0.1 mg/kg, i.p.	Male C57BL/6 mice	-	TST	Decreased immobility	[106]
	Acute, 1, 3.2 mg/kg, i.p.	Male SD rats	-	FST	Decreased immobility	[105]
	Acute, 5, 10 ng, i.c.v.	NMRI mice	Methamphetamine	FST	Decreased immobility	[76]
	Acute, 0.05, 0.1, 1, 5, 10 μg, i.c.v.	Male Swiss mice	-	FST	Decreased immobility	[108]
	Acute, 0.01, 0.1, 1 nmol, vmPFC microinjection	Male Wistar rats	-	FST	Decreased immobility	[109]
	Acute, 0.01 μg, PFC microinjection	Male Wistar rats	-	FST	Decreased immobility	[91]
HU-210 (CB1/CB2 agonist)	Acute, 0.5, 1 μg, dentate gyrus microinjection	Male Wistar rats	-	FST	Decreased immobility	[110]
AM404 (AEA reuptake inhibitor)	Acute, 5 mg/kg, i.p.	Male Long-Evans rats Rats	-	FST	Decreased immobility	[88]
	Acute, 0.1, 0.3, 1, 3 mg/kg, i.p.		-	FST	Decreased immobility	[89]
	Acute, 1 mg/kg, i.p.	Male SD rats	-	FST	Decreased immobility	[105]
	Acute, 0.1, 1, 5, 10 μg, i.c.v.	Male Swiss mice	-	FST	Decreased immobility	[108]
CP55,940 (CB1/CB2 agonist)	Acute, 0.03, 0.1, 0.3 mg/kg, i.p.	Male Wistar rats	-	FST	Decreased immobility	[89]
Oleamide	Acute, 10, 20 mg/kg, i.p.	Male Swiss mice	-	FST	Decreased immobility	[104]

rTMS: repetitive transcranial magnetic stimulation; CUMS: chronic unpredictable mild stress; CMS: chronic mild stress; ELS: early life stress; CHPG: (RS)-2-chloro-5-hydroxyphenylglycine; TST: Tail suspension test; i.c.v.: intracerebroventricular; CMS: chronic mild stress; HIPP: hippocampus; NMRI: Naval Medical Research Institute; CCI: chronic constriction injury; NP: neuropathic pain; SPT: sucrose preference test; SaPT: saccharine preference test; SD: Sprague–Dawley; ACEA: arachidonyl-2-chloroethylamide.

**Table 4:** A summary of the effects of CBD on depression-like behavior in rodents.

CBD Administration	Animals	Stress	Model	Effect	Reference
Acute, 200 mg/kg, i.p.	Male Swiss Webster mice	-	FST	Decreased immobility	[133]
7-day, 100 mg/kg, i.p.	Male C57BL/6J mice	-	FST	Decreased immobility	[134]
7-day, 10, 30 mg/kg, i.p.	Male SD rats	-	FST	Decreased immobility	[135]
Sub-chronic, 30 mg/kg, i.p.	Male Wistar rats	Streptozotocin (diabetic)	FST	Decreased immobility	[136]
Chronic, 10 mg/kg, i.p.	Male Wistar rats	CUMS	SPT	Elevated sucrose preference	[137]
Acute, 30 mg/kg, oral	Male and female WKY rats	WKY (genetic model)	SaPT	Elevated saccharine preference	[138]
Acute, 30 mg/kg, oral	Male and female WKY and male FSL rats	WKY or FSL (genetic models)	FST	Decreased immobility	[138]
Acute, 30 mg/kg, oral	Male WKY rats	WKY (genetic model)	SaPT	Elevated saccharine preference	[39]
Acute, 10 mg/kg, i.p.	Male Swiss mice	-	FST	Decreased immobility	[139]
Acute, 7 mg/kg, i.p. (co-administered with fluoxetine)	Male Swiss mice	-	FST	Decreased immobility	[139]
Acute, 10 mg/kg, i.p.	Male Swiss mice	-	FST	Decreased immobility	[141]
Acute, 7 mg/kg, i.p. (co-administered with AzaD or RG108)	Male Swiss mice	-	FST	Decreased immobility	[141]
Acute, 10 mg/kg, i.p.	Male Swiss mice	-	FST	Decreased immobility	[140]
Chronic, 30 mg/kg, i.p.	Male Wistar rats	-	FST	Decreased immobility	[142]
Acute, 30 mg/kg, i.p.	Male Wistar rats	-	FST	Decreased immobility	[142]
Acute, 10 mg/kg, i.v.	Male ICR mice	CMS	FST	Decreased immobility	[143]
Acute, 100 mg/kg, oral	Male ICR mice	CMS	FST	Decreased immobility	[143]
Acute, 15, 30, 60 nmol, mPFC microinjection	Male Wistar rats	CCI injury (NP)	FST	Decreased immobility	[144]
Acute, 30 mg/kg, i.p.	Male Swiss mice	-	FST	Decreased immobility	[145]
7 day, 50 mg/kg, i.p.	Male C57BL6 mice	OBX	SPT	Elevated sucrose preference	[146]
Acute, 10, 30, 45, 60 nmol, vmPFC microinjection	Male Wistar rats	-	FST	Decreased immobility	[147]

rTMS: repetitive transcranial magnetic stimulation; CUMS: chronic unpredictable mild stress; CMS: chronic mild stress; ELS: early life stress; CHPG: (RS)-2-chloro-5-hydroxyphenylglycine; TST: Tail suspension test; i.c.v.: intracerebroventricular; CMS: chronic mild stress; HIPP: hippocampus; NMRI: naval medical research institute; CCI: chronic constriction injury; NP: neuropathic pain; SPT: sucrose preference test; SaPT: saccharine preference test; SD: Sprague–Dawley; ACEA: arachidonyl-2-chloroethylamide; WKY: Wistar Kyoto; FSL: Flinders Sensitive Line; i.v.: intravenous; OBX: olfactory bulbectomy.
